# Association of gender to outcome after out-of-hospital cardiac arrest – a report from the International Cardiac Arrest Registry

**DOI:** 10.1186/s13054-015-0904-y

**Published:** 2015-04-21

**Authors:** Viktor Karlsson, Josef Dankiewicz, Niklas Nielsen, Karl B Kern, Michael R Mooney, Richard R Riker, Sten Rubertsson, David B Seder, Pascal Stammet, Kjetil Sunde, Eldar Søreide, Barbara T Unger, Hans Friberg

**Affiliations:** Department of Clinical Sciences, Lund University, 22184 Lund, Sweden; Department of Anaesthesiology and Intensive Care, Skåne University Hospital, Lund, 22185 Sweden; Department of Anaesthesiology and Intensive Care, Helsingborg Hospital, 25187 Helsingborg, Sweden; Sarver Heart Center, University of Arizona, 1501 N Campbell Ave, Tucson, AZ 85724 USA; Minneapolis Heart Institute Foundation, Abbot Northwestern Hospital, 920 E 28th Street 100, Minneapolis, MN 55407 USA; Department of Critical Care Services and Neuroscience Institute, Maine Medical Center, 22 Bramhall Street, Portland, ME 04102 USA; Department of Surgical Sciences/Anaesthesiology and Intensive Care, Uppsala University, Akademiska sjukhuset, 75185 Uppsala, Sweden; Department of Anesthesia and Intensive Care, Centre Hospitalier de Luxembourg, 4, rue Barblé, L-1210 Luxembourg, Luxembourg; Department of Anaesthesiology, Surgical ICU Ullevål, Oslo University Hospital, Oslo, Norway; Department of Anaesthesiology and Intensive Care, Stavanger University Hospital, 4068 Stavanger, Norway; Department of Clinical Medicine, University of Bergen, Jonas Lies veg 87, 5021 Bergen, Norway

## Abstract

**Introduction:**

Previous studies have suggested an effect of gender on outcome after out-of-hospital cardiac arrest (OHCA), but the results are conflicting. We aimed to investigate the association of gender to outcome, coronary angiography (CAG) and adverse events in OHCA survivors treated with mild induced hypothermia (MIH).

**Methods:**

We performed a retrospective analysis of prospectively collected data from the International Cardiac Arrest Registry. Adult patients with a non-traumatic OHCA and treated with MIH were included. Good neurological outcome was defined as a cerebral performance category (CPC) of 1 or 2.

**Results:**

A total of 1,667 patients, 472 women (28%) and 1,195 men (72%), met the inclusion criteria. Men were more likely to receive bystander cardiopulmonary resuscitation, have an initial shockable rhythm and to have a presumed cardiac cause of arrest. At hospital discharge, men had a higher survival rate (52% vs. 38%, *P* <0.001) and more often a good neurological outcome (43% vs. 32%, *P* <0.001) in the univariate analysis. When adjusting for baseline characteristics, male gender was associated with improved survival (OR 1.34, 95% CI 1.01 to 1.78) but no longer with neurological outcome (OR 1.24, 95% CI 0.92 to 1.67). Adverse events were common; women more often had hypokalemia, hypomagnesemia and bleeding requiring transfusion, while men had more pneumonia. In a subgroup analysis of patients with a presumed cardiac cause of arrest (n = 1,361), men more often had CAG performed on admission (58% vs. 50%, *P* = 0.02) but this discrepancy disappeared in an adjusted analysis.

**Conclusions:**

Gender differences exist regarding cause of arrest, adverse events and outcome. Male gender was independently associated with survival but not with neurological outcome.

## Introduction

Out-of-hospital cardiac arrest (OHCA) is a major cause of death in the industrialised world. An estimated 275,000 Europeans have an OHCA each year and less than 10% survive to hospital discharge [1]. We know from previous studies that more OHCA patients are men [2,3], but reports on gender differences in OHCA outcome are conflicting, partly due to differences in the studies’ inclusion criteria. While a larger proportion of women with OHCA seem to be admitted to hospital alive [4-9], women have been reported to have a survival rate equal to or lower compared to men [4-8,10-12]. After adjusting for known predictors of outcome, some authors have concluded that female gender is independently associated with improved survival [6,7,13], while others have reported equal [4,5,11] or worse survival rates [2,14]. In addition, a lower quality of life among female OHCA survivors has been reported [15].

Male and female OHCA patients differ in baseline characteristics, as males are younger, and more likely to have a witnessed arrest or receive bystander cardiopulmonary resuscitation (CPR) [4-8,10-13], factors associated with a favourable outcome. Additionally, men more often have an arrest of cardiac etiology and more often have ventricular fibrillation (VF) or pulseless ventricular tachycardia (VT) as the initial cardiac rhythm [5,7,10,13,16], while non-cardiac etiologies like pulmonary embolism, intoxication and obstructive pulmonary disease seem to be more common among women [7,11,17,18]. Little is known about gender differences regarding cardiac interventions and the adverse events profile in OHCA patients in the intensive care unit (ICU).

The aim of this large observational study was to investigate the association of gender to outcome, coronary angiography (CAG) and adverse events in comatose OHCA patients treated with mild induced hypothermia (MIH). We hypothesised that female gender was associated with worse in-hospital outcome when adjusting for baseline differences.

## Material and methods

### Study population

The International Cardiac Arrest Registry (INTCAR) is a prospectively recorded multinational registry offering detailed description of the treatment and outcomes of cardiac arrest patients with return of spontaneous circulation (ROSC). The registry is ICU-based and predominantly includes patients treated with MIH.

This retrospective observational study includes a convenience sample of adult (≥18 years) OHCA patients treated with MIH at 45 cardiac arrest centres in 11 countries in Europe and the USA from 2006 to 2012. Some centres did not participate during the whole study period. Participating centres were asked to prospectively enter data on consecutive cardiac arrest patients with ROSC who were admitted to an ICU. Each centre treated their patients according to local protocols, using cooling devices of their choice. MIH was defined as a target temperature of 32 to 34°C in 99% of centres, a majority kept patients at the target temperature for 24 hours.

The cause of arrest was defined as cardiac, non-cardiac, or traumatic. An arrest was presumed to be of cardiac origin if no other cause was obvious or likely, according to the Utstein definitions [19]. Patients with a presumed traumatic cardiac arrest were excluded from the present study, as were patients with a cerebral performance category (CPC) of 3 or 4 prior to arrest, and patients with missing data on gender, outcome, arrest location, presumed cause of arrest, or CPC prior to arrest.

Local ethical approval was granted following the regulations of each participating hospital. The study was approved by the ethical review board in Lund, Sweden (272/2007). No consent was needed but information about the study was provided to patients who regained consciousness.

### Data collection and definitions

Utstein-style data on patient characteristics and cardiac arrest factors [19] was entered into an electronic case report form. Cardiac arrest data were retrieved from emergency services and ambulance records. Data on cardiac interventions and ICU management, including do-not-resuscitate orders (DNR orders) and decisions on withdrawal of life-sustaining therapy (WLST), were entered into the same registry. The database included an automatic range check. On-site monitoring was not performed.

Comorbidities were registered if they were pharmacologically or previously surgically treated, or subject to continuous supervision at the time of cardiac arrest. Adverse events during ICU care were recorded according to a predefined protocol. Bleeding requiring transfusion and intracerebral bleeding were registered as adverse events. Diagnosis of pneumonia was based on a new or progressive consolidation on chest X-ray combined with at least two of the following three findings: fever, leucocytosis, and purulent tracheal secretions. Severe sepsis and septic shock were defined according to the criteria of the American College of Chest Physicians and Society of Critical Care Medicine [20]. Arrhythmias were characterised as ventricular tachycardia, ventricular fibrillation, atrial fibrillation, tachycardia (>130 beats/min) or bradycardia (<40 beats/min). Metabolic and electrolyte disorders included sustained hyperglycemia (>8 mmol/L for ≥4 hours), hypoglycemia (<3 mmol/L), hypokalemia (<3 mmol/L), hypophosphatemia (<0.7 mmol/L), and hypomagnesemia (<0.7 mmol/L). Presence of seizures was based on clinical detection and diagnosis. A body temperature exceeding 38°C at any point during the first seven days after cardiac arrest was registered as an adverse event.

### Outcomes

Survival at hospital discharge was the primary outcome. Secondary outcomes were neurological outcome at hospital discharge, utilization of CAG on admission and the frequency of adverse events during ICU care. Neurological outcome was assessed using the CPC scale [21]. The scores of the CPC scale are: CPC 1 (normal function or minor disability), CPC 2 (moderate neurological disability but able to complete activities of daily living), CPC 3 (severe neurological impairment but conscious), CPC 4 (vegetative state or coma) and CPC 5 (death). A CPC score of 1 or 2 at hospital discharge was considered a good neurological outcome.

### Statistical analysis

Continuous variables are presented with median and interquartile range (IQR). Groups that were not normally distributed were compared using the Mann-Whitney *U* test. Binary outcomes are presented as counts and percentages and were analysed using the chi-square test. All *P* values are two-tailed, and a *P* <0.05 was considered significant.

To establish the association of gender to survival and neurological outcome, a logistic regression model was created. The model adjusted for gender, age, witnessed arrest, bystander CPR, time to ROSC, initial shockable rhythm, presumed cardiac cause of arrest and for the comorbidities that differed in frequency between the gender groups (significance level *P* <0.20), which were coronary disease, chronic obstructive pulmonary disease (COPD), neurological disease, obesity (body mass index >35 kg/m^2^), insulin-dependent diabetes mellitus and alcohol or drug abuse.

Another logistic regression model was established to investigate the association of gender to CAG on hospital admission in the subgroup of patients with a presumed cardiac cause of arrest. This model adjusted for circulatory shock on admission, initial shockable rhythm and ST-elevation myocardial infarction (STEMI), based on previous reports [22,23].

Goodness of fit for the logistic regression models was assessed using the Hosmer-Lemeshow test and an adequate fit was assumed if *P* >0.05. Odds ratios (ORs) reflect the odds for CAG, for survival to hospital discharge and for a good neurological outcome at hospital discharge, respectively. Statistical analyses were performed using IBM SPSS Statistics for Windows, Version 22.0. Armonk, NY: IBM Corp.

## Results

### Baseline and cardiac arrest characteristics

**Figure 1:**
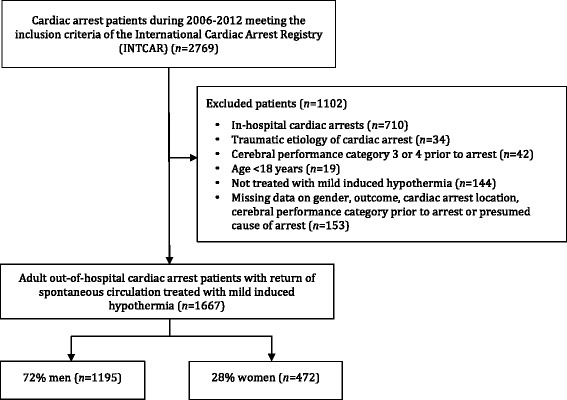
The International Cardiac Arrest Registry (INTCAR).

Over the six-year study period, 2,769 cardiac arrest patients with ROSC were registered in INTCAR. After exclusions, a total of 1,667 patients, 472 women (28%) and 1,195 men (72%), were eligible for inclusion (Figure 1). The median number of patients per site was 18 (range 1 to 232) and the number of included patients was similar in the United States (51%) and Europe (49%).

Patient characteristics and a comparison by gender are presented in Table 1. Men and women were pre-arrest healthy to the same degree but had different comorbidities with more coronary disease among men and more COPD among women. Despite no differences in the frequency of witnessed arrests, men were more likely to receive bystander CPR (64% vs. 58%, *P* = 0.03). Male patients were also more likely to have a presumed cardiac cause of arrest (86% vs. 71%, *P* <0.001), have a STEMI (32% vs. 21%, *P* <0.001), an initial shockable rhythm (69% vs. 52%, *P* <0.001) and to receive defibrillation (80% vs. 64%, *P* <0.001).

**Table 1:** **Baseline characteristics for total sample and by gender**

**Characteristic**	**Total,** ***n =*** **1,667**	**Men,** ***n =*** **1,195**	**Women,** ***n =*** **472**	***P***
Age, years (IQR)	62 (53–72)	63 (53–72)	62 (51–72)	0.22
Smoking, *n* = 1,278	579 (45)	419 (46)	160 (44)	0.47
Comorbidities				
Previously healthy	315 (19)	231 (19)	84 (18)	0.47
Coronary disease	560 (34)	425 (36)	135 (29)	0.01^*^
Heart failure	287 (17)	206 (17)	81 (17)	0.97
Cardiac arrhythmia	205 (12)	147 (12)	58 (12)	0.99
Arterial hypertension	780 (47)	560 (47)	220 (47)	0.93
Chronic obstructive pulmonary disease	221 (13)	127 (11)	94 (20)	<0.001^*^
Chronic renal failure	96 (6)	70 (6)	26 (6)	0.78
Neurological disease	142 (9)	92 (8)	50 (11)	0.06
Disseminated malignancy	53 (3)	37 (3)	16 (3)	0.76
Alcohol or drug abuse	212 (13)	162 (14)	50 (11)	0.10
Obesity (body mass index >35)	161 (10)	105 (9)	56 (12)	0.06
Diabetes (insulin dependent)	113 (7)	74 (6)	39 (8)	0.13
Diabetes (noninsulin dependent)	214 (13)	152 (13)	62 (13)	0.82
Cerebral performance category prior to arrest				
1	1,569 (94)	1,132 (95)	437 (93)	0.09
2	98 (6)	63 (5)	35 (7)	0.09
Witnessed arrest	1,369 (82)	994 (83)	375 (80)	0.07
Bystander CPR	1,030 (63)	758 (64)	272 (58)	0.03*
Initial cardiac rhythm				
Ventricular tachycardia/ventricular fibrillation	1,069 (64)	824 (69)	245 (52)	<0.001^*^
Pulseless electrical activity	212 (13)	124 (10)	88 (19)	<0.001^*^
Asystole	320 (19)	197 (17)	123 (26)	<0.001^*^
Unknown	66 (4)	50 (4)	16 (3)	0.45
STEMI	471 (29)	372 (32)	99 (21)	<0.001^*^
Cardiac cause of arrest	1,361 (82)	1,024 (86)	337 (71)	<0.001^*^
Non-cardiac cause of arrest	306 (18)	171 (14)	135 (29)	<0.001^*^
Defibrillation performed	1,261 (76)	959 (80)	302 (64)	<0.001^*^
Circulatory shock at hospital admission	475 (29)	325 (27)	150 (32)	0.07
Time from arrest to CPR by medical personnel, minutes (IQR), *n =* 1541	8 (5–12)	8 (5–12)	8 (5–13)	0.15
Time from arrest until ROSC, minutes (IQR), *n =* 1566	21 (14–33)	21 (13–33)	21 (14–32)	0.73

Data are presented as n (%) or median (IQR). When not stated otherwise, variables contain valid data for >95% of the 1,667 cases. For variables missing data from >5% of cases, *n* of valid cases is denoted. ^*^ *P* <0.05. CPR, cardiopulmonary resuscitation; IQR, interquartile range; ROSC, return of spontaneous circulation; STEMI, ST-elevation myocardial infarction.

### Survival and neurological outcome

The overall survival at hospital discharge was 48%. Table 2 shows outcome at discharge from ICU and hospital for total sample and by gender. Compared with women, men had significantly higher survival rates both at ICU discharge (59% vs. 46%, *P* <0.001) and at hospital discharge (52% vs. 38%, *P* <0.001). Males also had a higher rate of good neurological outcome at hospital discharge (43% vs. 32%, *P* <0.001). There were no gender differences in the length of ICU or hospital stay.

**Table 2:** **Outcome at discharge from ICU and hospital for total sample and by gender**

**Outcome**	**Total,** ***n =*** **1,667**	**Men,** ***n =*** **1,195**	**Women,** ***n =*** **472**	***P***
Alive at ICU discharge, *n* = 1,667	916 (55)	700 (59)	216 (46)	<0.001^*^
Cerebral performance category at ICU discharge, *n* = 1,667				
1	308 (19)	245 (21)	63 (13)	<0.001^*^
2	260 (16)	200 (17)	60 (13)	0.04^*^
3	163 (10)	122 (10)	41 (9)	0.35
4	185 (11)	133 (11)	52 (11)	0.95
5	751 (45)	495 (41)	256 (54)	<0.001^*^
Length of ICU stay, days (IQR)				
Patients alive at ICU discharge, *n* = 911	6 (4–10)	6 (4–10)	7 (4–9)	0.97
Patients dead at ICU discharge, *n* = 738	4 (2–6)	3 (2–5)	4 (2–6)	0.34
Alive at hospital discharge, *n* = 1,667	803 (48)	622 (52)	181 (38)	<0.001^*^
Good neurological outcome, *n* = 1,667	668 (40)	519 (43)	149 (32)	<0.001^*^
Cerebral performance category at hospital discharge, *n* = 1,667				
1	484 (29)	382 (32)	102 (22)	<0.001^*^
2	184 (11)	137 (12)	47 (10)	0.38
3	77 (5)	63 (5)	14 (3)	0.04^*^
4	58 (4)	40 (3)	18 (4)	0.64
5	864 (52)	573 (48)	291 (62)	<0.001^*^
Length of hospital stay, days (IQR)				
Patients alive at hospital discharge, *n* = 770	13 (9–21)	13 (9–21)	14 (9–20)	0.95
Patients dead at hospital discharge, *n* = 845	4 (2–7)	4 (2–7)	4 (2–7)	0.75

Data are presented as n (%) or median (IQR). *n* denotes the number of cases with valid data. ^*^ *P* <0.05. A cerebral performance category of 1 or 2 at hospital discharge is considered a good neurological outcome. ICU, intensive care unit; IQR, interquartile range.

### Multivariate analysis: association of gender to outcome

Table 3 shows the results of the multivariate logistic regression analysis. When adjusting for selected baseline characteristics, male gender was significantly associated with survival at hospital discharge (OR 1.34, 95% confidence interval (CI) 1.01 to 1.78) but not with a good neurological outcome (OR 1.24, 95% CI 0.92 to 1.67). Insulin-dependent diabetes and COPD were associated with lower survival but not with neurological outcome.

**Table 3:** **Multivariate logistic regression analysis of baseline factors and their association with outcome**

**Factor**	**Survival to hospital discharge**			**Good neurological outcome**		
	**OR**	***P***	**95% CI**	**OR**	***P***	**95% CI**
Male gender	1.34	0.04^*^	1.01–1.78	1.24	0.16	0.92–1.67
Age (per year)	0.96	<0.001^*^	0.95–0.97	0.96	<0.001^*^	0.95–0.97
Witnessed arrest	1.88	<0.001^*^	1.34–2.66	2.30	<0.001^*^	1.57–3.37
Bystander CPR	1.29	0.06	0.99–1.66	1.28	0.08	0.98–1.67
Presumed cardiac cause of arrest	2.16	<0.001^*^	1.40–3.31	2.51	<0.001^*^	1.54–4.08
Time to ROSC (per minute)	0.95	<0.001^*^	0.95–0.96	0.95	<0.001^*^	0.94–0.96
Initial shockable rhythm	3.95	<0.001^*^	2.90–5.38	4.84	<0.001^*^	3.41–6.88
Comorbidities						
Coronary disease	0.90	0.46	0.69–1.19	0.86	0.30	0.65–1.14
COPD	0.68	0.049^*^	0.47–1.00	0.71	0.11	0.47–1.08
Neurological disease	0.63	0.045^*^	0.40–0.99	0.57	0.03^*^	0.35–0.93
Obesity (body mass index > 35)	0.69	0.08	0.45–1.05	0.65	0.06	0.42–1.02
Diabetes (insulin dependent)	0.59	0.04^*^	0.36–0.98	0.81	0.42	0.48–1.37
Alcohol or drug abuse	0.98	0.92	0.66–1.45	0.92	0.69	0.61–1.39

A total of 1,494 of the 1,667 patients had complete data and were included in the analysis. A cerebral performance category of 1 or 2 at hospital discharge is considered a good neurological outcome. ^*^ *P* <0.05. CI, confidence interval; COPD, chronic obstructive pulmonary disease CPR, cardiopulmonary resuscitation; ROSC, return of spontaneous circulation; OR, odds ratio.

### Do-not-resuscitate orders and withdrawal of life-sustaining therapy

Among the 864 patients who did not survive until hospital discharge, 573 men (66%) and 291 women (34%), there was no gender difference in the frequency of DNR orders (women 76% vs. men 76%, *P* = 0.85) or deaths after WLST (women 81% vs. men 76%, *P* = 0.07).

### Coronary angiography and percutaneous coronary intervention in patients with a presumed cardiac cause of arrest

Among 1,361 patients, 1,024 men (75%) and 337 women (25%) with a presumed cardiac cause, a larger proportion of male patients had CAG on hospital admission (58% vs. 50%, *P* = 0.02). Among those, 62% male and 50% female (*P* = 0.01) received subsequent percutaneous coronary intervention (PCI). In a multivariate logistic regression model including 1,278 of the 1,361 patients with complete data, male gender was not independently associated with CAG on admission (OR 1.16, 95% CI 0.87 to 1.54). Patients who received PCI on admission had higher survival rates (61% vs. 50%, *P* <0.001) and better neurological outcome (52% vs. 42%, *P* <0.001).

### Adverse events in the intensive care unit

Men and women had different adverse events profiles during ICU care. The frequencies of adverse events for the total sample and by gender are shown in Table 4. Women were more likely than men to have bleeding requiring transfusion (11% vs. 8%, *P* = 0.048), hypokalemia (33% vs. 23%, *P* <0.001) or hypomagnesemia (28% vs. 23%, *P* = 0.02). Men were more likely to have pneumonia (40% vs. 32%, *P* <0.001) and to have a body temperature exceeding 38°C during the first seven days after the cardiac arrest (38% vs. 30%, *P* = 0.01).

**Table 4:** **Adverse events during ICU care for total sample and by gender**

**Adverse event**	**Total,** ***n =*** **1,667**	**Men, n = 1,195**	**Women, n = 472**	***P***
Bleeding				
Bleeding requiring transfusion	144 (9)	93 (8)	51 (11)	0.048^*^
Intracerebral bleeding	13 (1)	9 (1)	4 (1)	0.84
Infection				
Pneumonia	634 (38)	483 (40)	151 (32)	<0.001^*^
Severe sepsis/septic shock	131 (8)	95 (8)	36 (8)	0.83
Other infection	54 (3)	33 (3)	21 (4)	0.08
Arrhythmia				
Bradycardia (<40 beats/min)	262 (16)	195 (16)	67 (14)	0.28
Tachycardia (>130 beats/min)	82 (5)	60 (5)	22 (5)	0.76
Atrial fibrillation	208 (13)	155 (13)	53 (11)	0.33
Ventricular tachycardia	179 (11)	132 (11)	47 (10)	0.52
Ventricular fibrillation	131 (8)	100 (8)	31 (7)	0.22
Other arrhythmia	73 (4)	47 (4)	26 (6)	0.16
Metabolic or electrolyte disorder				
Hypoglycemia (<3 mmol/L)	58 (4)	38 (3)	20 (4)	0.29
Sustained hyperglycemia (>8 mmol/L >4 hrs)	884 (53)	625 (52)	259 (55)	0.34
Hypokalemia (<3 mmol/L)	433 (26)	278 (23)	155 (33)	<0.001^*^
Hypomagnesemia (<0.7 mmol/L)	405 (24)	272 (23)	133 (28)	0.02^*^
Hypophosphatemia (<0.7 mmol/L)	331 (20)	241 (20)	90 (19)	0.61
Clinical seizures	443 (27)	314 (26)	129 (27)	0.66
Body temperature exceeding 38°C during the first 7 days after cardiac arrest	582 (36)	441 (38)	141 (30)	0.01^*^

Data are presented as n (%). Variables contain valid data for >95% of the 1,667 cases. ^*^ *P* <0.05. ICU, intensive care unit.

## Discussion

The main result of this large registry study is that men had a higher survival rate and a better neurological outcome at hospital discharge. When adjusting for differences in baseline characteristics, male gender was independently associated with survival at hospital discharge but not with neurological outcome. We also identified gender differences in the frequencies of adverse events in the ICU. In a subgroup analysis of patients with a presumed cardiac cause of arrest, men more often had CAG on admission, but gender was not independently associated with CAG in an adjusted model.

This large registry study involving sites in Europe and the United States adds important information on gender differences after OHCA, many of which have previously been reported [4,5,7,10,11,13]. Our study differs from most previous studies since we only included patients who achieved ROSC and who were admitted to an ICU where they received MIH, which allowed us to analyse the in-hospital mortality, frequency of interventions and adverse events. This difference between study populations makes a comparison of our results to many previous reports on gender differences after OHCA challenging. The overall survival rate in our study, however, was equal to or higher than what has been reported by others in comparable populations [2,3,24]. The proportion of male patients reported here is consistent with several previous studies [2,3], confirming that OHCA is more common among men, even though a recent systematic review reported a higher proportion of female patients [8].

It has been reported that female gender, when adjusted for known predictors of outcome, is associated with improved survival to hospitalization but with equal or lower survival to hospital discharge [4,5,11]. The reason for the higher in-hospital mortality for women can only be speculated on but is in line with the findings of the present study. One explanation is the difference in baseline characteristics, such as the rate of bystander CPR, witnessed arrests and in the location and cause of arrest [5-7]. Increasing age was a significant predictor of poor outcome in our study as in previous studies, but we could not detect any gender differences as regards age [2,13]. Nor could we identify a gender difference in the frequency of DNR orders or WLST that could explain the increased in-hospital mortality among women. There have been reports of a survival benefit for women of reproductive age after both OHCA [16,25] and in-hospital cardiac arrest [26], suggesting a protective effect of endogenous estrogen. Unfortunately, we could not investigate this aspect in the present study due to the limited number of young female patients.

Previous studies have shown that men more often have an OHCA in a public place while women more often arrest in their home, which may explain the higher frequency of witnessed arrests and bystander CPR in male patients [5,7,12]. Whether this is true in the present study is not known since information on location of arrest in the INTCAR is lacking. There was, however, no significant difference in the proportion of patients with witnessed arrest. In spite of that, significantly more male patients received bystander CPR. Possible explanations are that our study was underpowered to detect an existing gender difference in witnessed arrests, that women are more prone to start CPR than men, or that bystanders are uncomfortable providing CPR to women. The higher frequency of bystander CPR among male patients may explain why they more often were found in shockable rhythms [27,28]. Another explanation for the increased rate of shockable rhythm in male patients may be that men more often have cardiac arrest of cardiac etiology [7,10], which is supported by our findings.

It is well known that medical comorbidities are common among OHCA patients [3,24]. We found that men and women were previously healthy to the same degree, but that men had more coronary disease while women had more COPD. This gender difference is consistent with findings from a recent prehospital study [12] and may have contributed to the higher rate of a non-cardiac cause of arrest and concurrent lower survival in female patients in our study [17].

Numerous studies on patients with cardiovascular disease have reported a male dominance in the utilization of interventional cardiac procedures [29-32], but gender differences regarding CAG/PCI after OHCA has, to our knowledge, not previously been studied. Early CAG/PCI is established as standard treatment for OHCA patients with STEMI, but it is less clear if early CAG/PCI is beneficial in patients without STEMI [33,34]. Since ST-elevation is rarely seen and chest pain can often not be assessed in OHCA patients [23], guidelines prescribe that CAG/PCI should be considered in all cardiac arrest patients with suspected coronary artery disease [35]. A recent systematic review and meta-analysis concluded that early CAG after OHCA was associated with improved outcome and better survival, though randomised clinical trials are lacking [36]. We found that a higher proportion of men with a presumed cardiac cause of arrest had CAG on hospital admission, but male gender was not an independent predictor of CAG when adjusted for differences in rates of STEMI, initial shockable rhythm and circulatory shock on admission. Among patients receiving angiography, more men than women had PCI performed, which is in accordance with a previous study [2]. The reason for this discrepancy can only be speculated on and may be a matter of chance, or possibly due to increased complexity in assessing suspected coronary disease after cardiac arrest in female patients.

We present for the first time detailed data on gender differences in the frequency and profile of adverse events during ICU care that potentially could have a positive effect on the daily management of OHCA patients. Pneumonia in the ICU has previously been described as an independent predictor of survival after OHCA, probably because survivors stay longer in the ICU and therefore have time to develop infections [37]. We found that men more often were diagnosed with pneumonia, but surprisingly no gender difference in the length of ICU stay. It is likely that the higher frequency of fever in the ICU among men reflects their higher rate of pneumonia. We also found that women had more bleeding requiring transfusion and more electrolyte disorders, like hypokalemia and hypomagnesemia during ICU care. A recent study showed that hypokalemia was associated with increased mortality after OHCA, a finding that disappeared when adjusted for other predictors of outcome [37]. From our study, we cannot determine if the gender disparities in adverse events indicate differences in the severity of illness, in medication, or if it may be a sign of a gender difference in the quality of post-arrest care.

A more speculative explanation for the gender differences reported here is that in a recent study, women were less likely to benefit from hypothermia treatment after OHCA [38]. In light of the recently published Target Temperature Management after cardiac arrest trial (TTM trial), this explanation seems less probable [24].

### Limitations

There are a number of limitations to this study. This is a retrospective study of prospectively collected registry data and the sample size was determined not by a calculation of power but by convenience. No audit or formal quality control was performed, making erroneous data entries possible. The generalisability of our findings may be disputed, as our results reflect standards in highly specialised OHCA centres using MIH. Hospital characteristics are known to predict OHCA outcome, favouring centres with 24-hour cardiac interventional services [14]. Also, we cannot exclude that the association between male gender and survival was a spurious finding, given the CIs. Finally, we report no data on long-term outcome for the patients. A recent study, however, showed that the difference between hospital survival and 180-day survival is limited [24].

## Conclusions

In this large registry study of comatose OHCA patients treated with MIH, we identified gender differences in cause of arrest, initial rhythm, bystander CPR, adverse events and outcome. After adjustment for baseline differences, male gender was associated with improved survival but not with neurological outcome. Among patients with a presumed cardiac cause of arrest, a larger proportion of men had CAG on hospital admission, but this difference disappeared in an adjusted model. There was no gender difference in the frequency of DNR orders and WLST in deceased patients.

## Key messages

- When adjusting for differences in baseline characteristics, male gender was independently associated with survival to hospital discharge but not with neurological outcome.
- In patients with a cardiac cause of arrest, more men than women had coronary angiography on admission, but this difference disappeared when adjusting for differences in STEMI, circulatory shock and initial shockable rhythm.
- The adverse event profile differed during ICU care; women more often had hypokalemia, hypomagnesemia and bleeding requiring transfusion, while men had more pneumonia.

## Abbreviations

CAG: coronary angiography
CI: confidence interval
COPD: chronic obstructive pulmonary disease
CPC: cerebral performance category
CPR: cardiopulmonary resuscitation
DNR order: do-not-resuscitate order
ICU: intensive care unit
INTCAR: International Cardiac Arrest Registry
IQR: interquartile range
MIH: mild induced hypothermia
OHCA: out-of-hospital cardiac arrest
OR: odds ratio
PCI: percutaneous coronary intervention
ROSC: return of spontaneous circulation
STEMI: ST-elevation myocardial infarction
VF: ventricular fibrillation
VT: ventricular tachycardia
WLST: withdrawal of life-sustaining therapy
